# Effects of Simulated Microgravity on Human Umbilical Vein Endothelial Cell Angiogenesis and Role of the PI3K-Akt-eNOS Signal Pathway

**DOI:** 10.1371/journal.pone.0040365

**Published:** 2012-07-13

**Authors:** Fei Shi, Yong-Chun Wang, Tian-Zhi Zhao, Shu Zhang, Ting-Yuan Du, Chang-Bin Yang, Ying-Hui Li, Xi-Qing Sun

**Affiliations:** 1 Department of Aerospace Biodynamics, Fourth Military Medical University, Xi’an, Shaanxi, China; 2 State Key Laboratory of Space Medicine Fundamentals and Application, Chinese Astronaut Research and Training Center, Beijing, China; University of Florida, United States of America

## Abstract

Endothelial cells are very sensitive to microgravity and the morphological and functional changes in endothelial cells are believed to be at the basis of weightlessness-induced cardiovascular deconditioning. It has been shown that the proliferation, migration, and morphological differentiation of endothelial cells play critical roles in angiogenesis. However, the influence of microgravity on the ability of endothelial cells to foster angiogenesis remains to be explored in detail. In the present study, we used a clinostat to simulate microgravity, and we observed tube formation, migration, and expression of endothelial nitric oxide synthase (eNOS) in human umbilical vein endothelial cells (HUVEC-C). Specific inhibitors of eNOS and phosphoinositide 3-kinase (PI3K) were added to the culture medium and gravity-induced changes in the pathways that mediate angiogenesis were investigated. After 24 h of exposure to simulated microgravity, HUVEC-C tube formation and migration were significantly promoted.This was reversed by co-incubation with the specific inhibitor of N-nitro-L-arginine methyl ester hydrochloride (eNOS). Immunofluorescence assay, RT-PCR, and Western blot analysis demonstrated that eNOS expression in the HUVEC-C was significantly elevated after simulated microgravity exhibition. Ultrastructure observation via transmission electron microscope showed the number of caveolae organelles in the membrane of HUVEC-C to be significantly reduced. This was correlated with enhanced eNOS activity. Western blot analysis then showed that phosphorylation of eNOS and serine/threonine kinase (Akt) were both up-regulated after exposure to simulated microgravity. However, the specific inhibitor of PI3K not only significantly downregulated the expression of phosphorylated Akt, but also downregulated the phosphorylation of eNOS. This suggested that the PI3K-Akt signal pathway might participate in modulating the activity of eNOS. In conclusion, the present study indicates that 24 h of exposure to simulated microgravity promote angiogenesis among HUVEC-C and that this process is mediated through the PI3K-Akt-eNOS signal pathway.

## Introduction

It has been demonstrated that exposure to microgravity during space expeditions can induce many of changes in human physiological systems.These include loss of bone mineral density, muscle atrophy, cardiovascular deconditioning, and impairment of pulmonary function [1]. The cardiovascular system is especially affected by space flight, with changes manifesting as cardiac dysrhythmias, cardiac atrophy, orthostatic intolerance, and reduced aerobic capacity [2].

The endothelium plays a pivotal role in maintaining microvascular homeostasis, regulating local blood flow, and other physiological processes [3]. Recent reports have indicated that endothelial cells (ECs) on the interior surfaces of vessels are highly sensitive to microgravity and undergo morphological and functional changes under these conditions [4]–[6]. Because ECs are key to vascular function, inflammation, and angiogenesis, endothelial dysfunction is likely to be one important factor of the basis of weightlessness-induced cardiovascular deconditioning [7], [8]. For this reason, several studies have been devoted to the means by which microgravity affects endothelial cell functions. These studies have used a variety of in vitrocell models.

Angiogenesis is the process by which new blood vessels form from pre-existing endothelial structures [9]. Previous studies have shown that endothelial cell proliferation, migration and morphological differentiation play critical roles in this process [10]. ECs are very heterogeneous, which is one reason why the influence of microgravity on ECs and angiogenesisis sodifficult to determine. Different behaviors have been observed in ECs cultured in modeled microgravity. Carlsson et al. found that the proliferation rate of endothelial cells to be increased under simulated microgravity [4]. Morbidelli et al. obtained opposite results in porcine aortic ECs [6]. The expression of many angiogenic molecules, such as nitric oxide (NO), vascular endothelial cell growth factor (VEGF) and endothelin-1 are modified under microgravity conditions, which also indicates that microgravity conditions may influence angiogenesis [5], [8], [11].

NO produced by endothelial NO synthase (eNOS) affects the regulation of vascular tone, vascular remodeling, and angiogenesis [12]–[14]. eNOS produces NO constitutively at low levels and can be transiently stimulated to produce high levels of NO by chemical agonists and by various mechanical forces. Growing amounts of evidence have established the involvement of NO in simulated microgravity-induced angiogenesis. NOS inhibitors block simulated microgravity-induced ECs migration, proliferation, and tube formation in vitro [15]. Many growth factors and hormones have been shown to induce phosphoinositide 3-kinase-Akt (PI3K-Akt)-dependent phosphorylation of eNOS, which activates NOS and induces subsequent increases in NO production [16], [17]. For example, in coronary arteries, Ang-1 has been shown to protect endothelial cells against oxidized low-density lipoprotein-induced injury via a PI3K-Akt-dependent mechanism [18]. Dimmeler et al. demonstrated that shear stress activates eNOS by phosphorylation of the enzyme through the PI3K-Akt pathway [19], [20]. However, thus far, little is known about the impact of microgravity on endothelial cell angiogenesis, migration, or the mechanisms underlying these processes.

Parabolic flight and freefall can be used to simulate microgravity [21]. However, the duration of microgravity conditions generated in these ways is usually too short to alter cell growth or differentiation. Currently, many efforts have been made toward establishing alternative methods of simulating microgravity conditions on Earth. Clinostats are considered reasonably effective ground-based tools for simulating microgravity [22], [23]. With the aim of better evaluating the pathophysiological modifications that microgravity induces in endothelial cells, we first hypothesized that the up-regulation of eNOS induced by simulated microgravity might be mediated via the PI3K-Akt pathway, thereby promoting the angiogenesis and migration of endothelial cells. In order to validate our hypothesis, we designed the current studies to determine the exact changes of endothelial cell angiogenesis and migration caused by simulated microgravity through in vitro tube formation and wound healing experiments. We then used many cell biology and molecular techniques to determine the underlying mechanisms.

## Materials and Methods

### Materials

Matrigel™ matrix (basement membrane) was obtained from BD Biosciences (Oxford, U.K.). eNOS inhibitor, N-nitro-L-arginine methylester hydrochloride (L-NAME) was obtained from Sigma. It was dissolved in water and stored at −70°C. LY294002 (PI3K inhibitor) was obtained from Cell Signaling, dissolved in DMSO, and stored at −20°C. Antibodies against eNOS, phospho-Akt (Ser^473^) (p-Akt), Akt, phospho-eNOS (Ser^1177^) (p-eNOS), and GAPDH were all from Cell Signaling Technology (Beverly, CA, U.S.). Alexa Fluor 488 Goat Anti-rabbit IgG and Hoechst 33258 were purchased from Sigma-Aldrich (St. Louis, MO, U.S.). Trizol reagent from Invitrogen Life Technologies (Carlsbad, CA, U.S.), PrimeScript® RT Reagent Kit and Premix Taq® Version 2.0 were obtained from TaKaRa Biotechnology Co. (Ohtsu, Shiga, Japan).

### Cell Culture Procedure

Human umbilical vein endothelial cells (HUVEC-C) were purchased from American Type Culture Collection (ATCC, Manassas, VA, U.S.) and cultured in an F-12K Medium (ATCC, U.S.) supplemented with 10% fetal bovine serum (Hyclone Laboratories, Logan, UT), 0.05 mg/ml endothelial cell growth supplement (ECGS), 0.1 mg/ml heparin, 100 units/ml penicillin and 100 mg/ml streptomycin (Sigma-Aldrich, St. Louis, MO, U.S.) under normal cell culture conditions (37°C, 5% CO_2_). The cells used in these experiments were generally from passages two to three.

### Simulation of Microgravity Conditions

**Figure 1:**
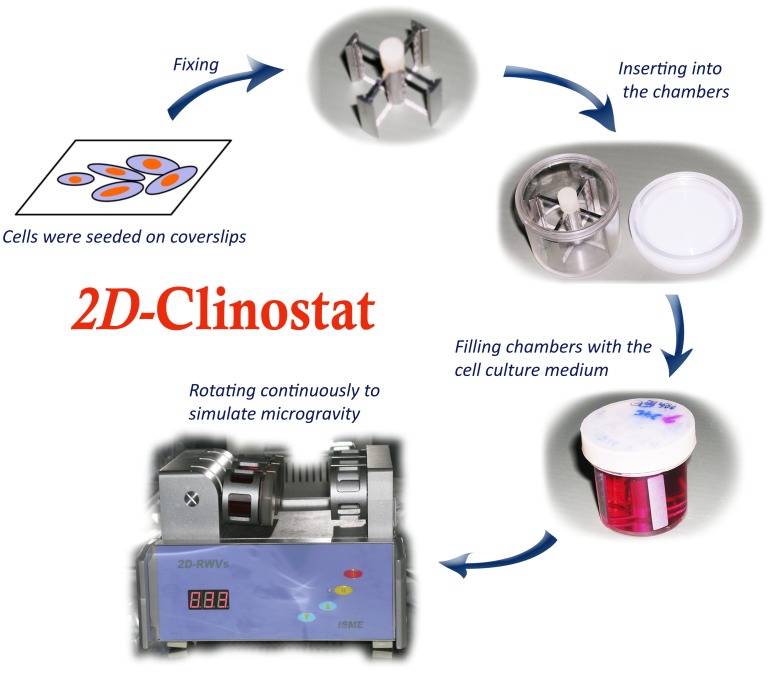
Setup of the clinostat model system. The clinostat is equipped with computerized temperature and motor controls. Cells were initially seeded on coverslips, and then the coverslips were inserted into the fixtures of the chambers. After that, the chambers were filled completely with F-12K with 10% FBS (filling chambers with complete medium, taking care to avoid air bubbles). These chambers underwent rotation at 30 rpm around the vertical axis for 24 h. NG controls were static control cultures kept in the same room as the clinostat.

The clinostat is an effective, ground-based tool that can be usually used to generate hypogravity. The weightlessness created by a clinostat is often regarded as simulated microgravity. Under these conditions, cells cannot feel gravity; the gravity vector escapes its detection machinery. The machines described are used under the assumption that the inability to sense gravity has effects similar to actual weightlessness. The clinostat model system (clinorotation) used in this study is an equipment for providing a vector-averaged reduction in the apparent gravity on the cell culture. On a space station or spacecraft, gravitational forces are approximately 10^−4^. The 2D-clinostat used in these experiments only models certain aspects of microgravity, and the average gravitational force acting on the cells is reduced to about 10^−3^ when the clinostat rotates at 30 rpm. However, these devices can be used to develop hypotheses concerning gravitational cell biology and to direct the design and scope of orbital flight studies. Our previous results documenting alterations in the function of HUVEC-C when cultured in the clinostat mimic the results obtained in true microgravity, suggesting the suitability of using these surrogate systems for bench-top microgravity research [24]. The clinostat and stimulated microgravity protocol are shown in Figure 1. The chambers were rotated around the horizontal axis.

Clinorotation experiments have been used to study the effects of simulated microgravity since 1965. When using the clinostat to simulate microgravity, it is important to solve the problems which affect the results of clinorotation experiments, including the shear stress and vibration generated by the clinostat. In the present study, we limited the shear stress by filling the chamber completely with the culture medium and invited a paralleled control to eliminated effects of vibrations. Cell rotary training was performed according to our routine protocols, as published previously [24]. Briefly, HUVEC-C were seeded at a density of 1×10^5^ cells on 2.55×2.15 cm coverslips. After the cells grew for 24 h and adhered to the coverslips, the coverslips were inserted into the slots of the stainless steel fixture which could be placed into the plastics vessel and were situated 12.5 mm from the rotational axis. After that, the chambers were filled completely with the culture medium containing the abovementioned supplements by pipette. Aspiration was performed gently to eliminate air bubbles. The assembled chambers were randomly divided into three groups: (1) cells exposed to normal gravity for 24 h (Cont), (2) cells exposed to simulated microgravity for 24 h (Clino), (3) cells exposed to simulated microgravity in the presence of inhibitors (100 µM L-NAME or 50 µM LY294002) for 24 h (Clino+L-NAME/Clino+LY294002). These chambers were then fixed onto the clinostat (2D-Clinostat, developed by China Astronaut Research and Training Center). The entire system was placed in a humidified incubator at 37°C under 5% CO_2_, and HUVEC-C of simulated microgravity groups were exposed to clinorotation at 30 rpm for 24 h. The NG controls were static control cultures kept in the same room as the clinostat.

### Inhibitor Study Protocol

A pharmacological inhibition study was performed to investigate the role of eNOS in microgravity-stimulated angiogenesis. L-NAME is a highly potent eNOS inhibitor. The HUVEC-C were treated with 100 µM L-NAME in the medium. HUVEC-C complete medium containing L-NAME was replenished the 42 ml-vessels that were inserted into a rotary clinostat. The control cells were cultured in the absence of the eNOS inhibitor. The involvement of PI3K-Akt in the increases in eNOS levels that may have been induced by simulated microgravity was tested by using the PI3K-inhibitor LY294002 (50 µM). LY294002-containing endothelial cell medium was added to clinorotation HUVEC-C according above-mentioned methods.

### Endothelial Cell Tube Formation Assay

The formation of tube-like structures by HUVEC-C on Matrigel was performed as previously described [25], [26]. The night before the tube formation assay, BD Matrigel matrix was incubated overnight on ice. On the day of the assay, 250 µl Matrigel matrix was transferred to a 24-well plate. It was allowed to polymerize at 37°C, 5% CO_2_ for 1 h. After rotation or static culture for 24 h, HUVEC-Cwere harvested at appropriate cell densities. Then 400 µl cells at 2×10^5^ ml in F-12K medium were seeded onto pre-solidified Matrigel in each well. Plates were incubated at 37°C for 20 h with 5% CO_2_. Tube formation was observed using an inverted phase contrast microscope. Images were captured with a video graphic system. The degree of tube formation at 20 h was quantified by measurement of the length of tubes in five randomly chosen low-power (40×) fields from each well using Image-Pro Plus software. The control sample was defined as 100% tube formation, and the increase or decrease in tube formation relative to the control was calculated for each sample. Each experiment was repeated at least three times under similar conditions.

### Cell Migration Assay

After culturing, the control and microgravity-exposed cell coverslips were taken out from the chambers and placed in 6-well plates. The wound migration assay was performed as described by Leavesley et al. with slight modifications [27]. At time zero, the cells were mechanically wounded by scraping with a 200 µl pipette tip, denuding a strip of the monolayer 300 µm wide. The boundary of the wound was marked. The cells were rinsed three times with PBS buffer to remove dislodged cells and cellular debris. F-12K medium containing 10% FBS was added and the wound was observed 24 h later with an Olympus microscope at 40× fitted with an ocular grid. Images were taken with a Nikon Coolpix camera under phase contrast microscope. We measured the width of each denuded area and found the average. The migration ability was calculated by the formula: 100% − (width _24 h_/width_ 0 h_) × 100%.

### Immunofluorescence and Confocal Microscopy

The coverslips were removed from the culture medium and washed with PBS. Cells were fixed with cold 3% paraformaldehyde for 20 min and then immersed in PBS for about 10 min, followed by permeabilization with 0.2% Triton-X in PBS for 5 min.They were blocked with 1% PBS-BSA for 1 h. The cells were then exposed to rabbit polyclonal anti-eNOS antibody (dilution 1∶200) for 2 h at room temperature. For a negative control, identical cells were exposed to PBS alone for the same period of time. Both sets of cells were then washed three times with PBS, before exposure to Alexa Fluor 488-conjugated goat anti-rabbit IgG (dilution 1∶200) for 1 h at room temperature. HUVEC-C nuclei were stained for 10 min with 0.2 µg/ml Hochest 33258. Slides were washed another three times with PBS and then postfixed with Vectashield mounting medium (Vector Labs Inc, Burlingame, CA, U.S.), covered with glass coverslips, and subjected to confocal microscopy. Images were collected on an Olympus IX2 microscope equipped with an Olympus FV1000 laser scanning confocal system and Olympus 10×/0.40 air and 60×/1.20 water emersion objectives (Olympus, America Inc., Center Valley, PA, U.S.). Alexa-modified antibodies were excited at 490 nm using an argon laser and detected with a 505–550 nm band pass filter. Hochest was excited at 346 nm and detected with a long-pass band filter 460 nm. All images were processed using Image-pro 3D Suite. Approximately 100–150 cells were analyzed per experimental condition.

### Transmission Electron Microscopy (TEM)

To directly examine caveolae microstructure and distribution, HUVEC-C were processed for TEM. Briefly, after fixation for 1 h with cold 3% glutaraldehyde in 0.1 M cacodylate buffer (pH 7.3) and after several washes in 0.2 M cacodylate, the cells were gently scraped from the glass slides and pelleted by centrifugation. Cell pellets were then postfixed with 2% osmium tetroxide in 0.1 m cacodylate for 1 h at 4°C, stained en bloc with 2% uranyl acetate for an additional 1 h. After three more washes in double-distilled water, the samples were dehydrated in a series of acetone solutions and embedded in Epon 812 according to the standard procedure. Ultrathin sections (70 nm) were prepared, stained with both uranyl acetate and lead citrate, and assessed using a Hitachi 7400 electron microscope (Hitachi, Tokyo, Japan), operated at an accelerating voltage of 80 kV. Random fields taken from individual endothelial cell samples (n = 15 for each group) were photographed at ×30,000. Caveolae associated with the plasma membrane were quantified by counting the number of distinct flask shaped, non-coated vesicles (50–90 nm in diameter) found on or within 100 nm of the plasma membrane.

### Reverse Transcription-polymerase Chain Reaction (RT-PCR)

To detect the changes of eNOS gene expressions after 24 hours of clinorotation stimulation, total RNA was extracted from the control and clinostat groups by Trizol reagent according to the manufacturer’s instructions. First-strand cDNA was synthesized from 100 ng of total RNA using a PrimeScript® RT reagent Kit. Aliquots of the cDNA products were used as templates for PCR amplification in an automated thermal cycler. Specific primers HUVEC-C eNOS (NM_000603.4), forward primer: 5′-AGATCACCGAGCTCTGCATT-3′, reverse primer: 5′-ATTTCCACTGCTGCCT.

TGTC-3′, product size: 400 bp; GAPDH (NM_002046.3) forward primer: 5′-GGGA.

AACTGTGGCGTGAT-3′, reverse primers: 5′-AAAGGTGGAGGAGTGGGT-3′, product size: 309 bp. RT-PCR runs were performed with an initial activation step of 3 min at 94°C followed by 35 routine cycles. Each cycle included incubations at 94°C for 30 s, 56°C for 50 s, and at 72°C for 1 min. One additional cycle of 72°C for 7 min was run to allow trimming of incomplete polymerizations. Control reactions were performed in the absence of cDNA. PCR products were separated on 2% agarose gels by electrophoresis and visualized by staining with ethidium bromide. Images were acquired by Labworks Image Acquisition and Analysis software and expressed as the eNOS/GAPDH ratio.

### Western Blot Analysis

After 24 h of treatment, cells from each group were scraped off the coverslips and lysed with the cell lysis buffer (Cell Signaling Technology, Beverly, CA, U.S.) containing 20 mM Tris pH 7.5, 150 mM NaCl, 1% Triton X-100, 2.5 mM sodium pyrophosphate, 1 mM EDTA, 1%Na_3_VO_4_, 0.5 µg/ml leupeptin, 1 mM PMSF. The lysates were centrifuged at 10,000×g at 4°C for 20 min to remove the insoluble material. Supernatants were collected, and the protein concentration was then measured with the BCA Protein Assay Reagent (Pierce, Rockford, IL, U.S.). Then equal amounts of protein (30 µg) from each sample were separated by 10% SDS-PAGE and transferred to polyvinylidene difluoride membrane (Millipore Co., Billerica, MA, U.S.) at 250 mA for 2 h at 4°C. The membranes were blocked for 1 h in 5% skim milk and then incubated overnight at 4°C with primary antibody against eNOS (1∶200), or phospho-eNOS (1∶500), or Akt (1∶1000), or phospho-Akt (1∶500), or GAPDH (1∶2000). After two 10 min washes in TBST, the membranes were incubated for 1.5 h at room temperature with horseradish peroxide-conjugated secondary antibody diluted 1∶400 (Santa Cruz Biotechnology, Delaware, CA, U.S.), before being developed using ECL Plus Western Blotting Detection Reagents (Amersham Biosciences, Piscataway, NJ, U.S.). Bands were quantified by scanning densitometry and analyzed using Image Quant software. All immunoblot analyses were repeated three timesunder similar conditions.

### Statistical Analysis

Multiple samples were collected during each experiment (n = 3–5) and at least three independent experiments were performed. All data are presented as the means ± SD of at least three independent experiments. Statistical comparisons between groups were performed by one-way ANOVA followed by Student’s t-test. A value of *P≤*0.05 was considered statisticallysignificant.

## Results

### Effects of Simulated Microgravity on Tube Formation by HUVEC-C in Matrigel

**Figure 2:**
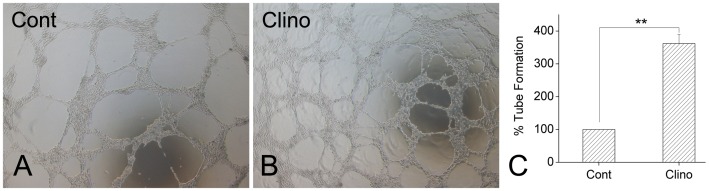
Effects of simulated microgravity on HUVEC-C tube formation. A: Representative images captured after incubation for 20 h. After cultured lasting 24 h, cells that had been incubated under static control conditions (Cont) or exposed to clinorotation (Clino) were seeded onto Matrigel. Tube-like structures appeared at 20 h as observed by phase contrast microscopy. B: Quantification of tube formation. Image-Pro Plus software was used to determine the total length of the tube-like structures in images. Histograms show tube formation as a percentage of that of control cells (n = 3, mean ± SD). ^**^ *P*<0.01 versus control.

To determine whether simulated microgravity affects the ability of HUVEC-C to form capillary-like tubes, HUVEC-C cultured under static control conditions and HUVEC-Cs exposed to clinorotation for 24 h were seeded in Matrigel and examined for tube formation microscopically. Between 1 and 2 h after plating, the HUVEC-C began to rearrange or align themselves in the control or clinorotation groups. By 8 h after plating, the clinorotation HUVEC-C had differentiated into an expansive and sharply defined tube network, but the majority of the control cells remained in individual clusters or ovoid colonies. At 20 h after plating, the clinorotation HUVEC-C showed a more extensive network of interconnecting tubes than control HUVEC-C (Figure 2A, 2B). To quantify tube formation, total tube length was calculated using Image-Pro Plus software. Clinorotation treatment increased HUVEC-C tube length by 257±21% over controls (Figure 2C). These findings suggest that simulated microgravity enhances the ability of HUVEC-C to form tube-like structures.

### Effects of L-NAME on the Promotion of HUVEC-C Tube Formation Induced by Simulated Microgravity

**Figure 3:**
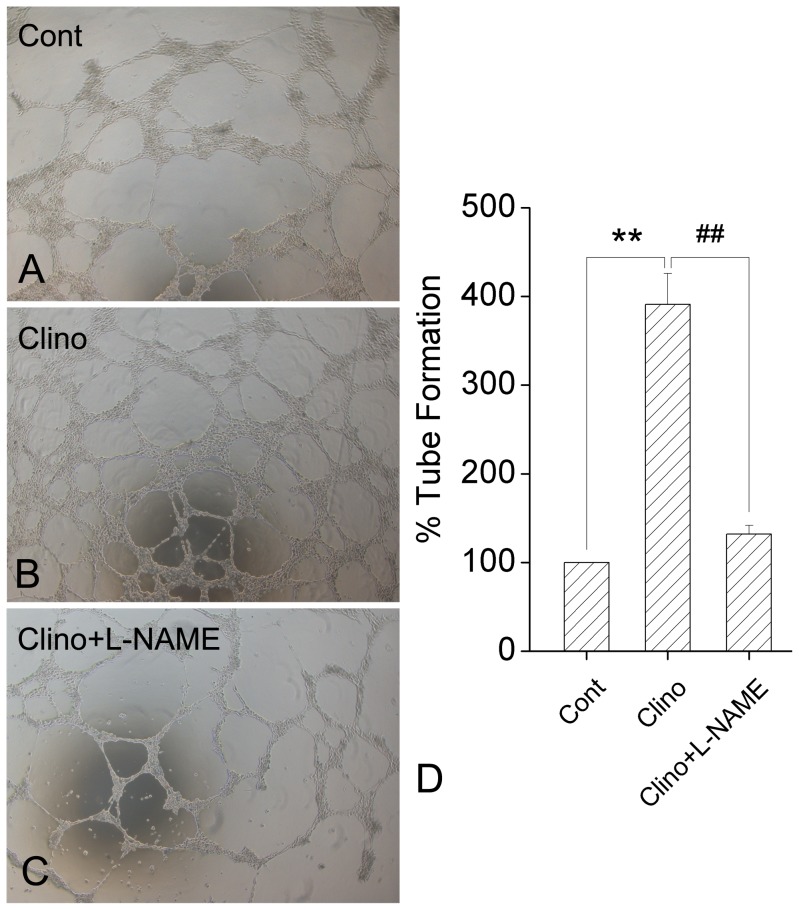
Effects of L-NAME on simulated-microgravity-induced tube formation in HUVEC-C. A. Tube-like structures of the Cont group, B. Tube-like structures of the Clino group, C. Tube-like structures of the Clino + L-NAME group. Shown are representative pictures of HUVEC-C plated on Matrigel for 20 h. D. Quantified tube formation as described in Figure 2B. Simulated microgravity significantly promoted the tube formation of HUVEC-C. Co-incubation with L-NAME, the specific inhibitor of eNOS, dramatically suppressed the HUVEC-C tube formation (n = 3, mean ± S.D.). ^**^ *P*<0.01 versus control group; ^##^ *P*<0.01 versus clinorotation group.

In order to determine whether eNOS is involved in angiogenesis promoted by simulated microgravity in HUVEC-C, L-NAME was added into the culture medium and its effects were examined through tube formation assay. We optimized the dose to prevent inhibition of basal HUVEC-C tube formation. The optimal concentration of L-NAME was 100 µM. HUVEC-C tube formation was dramatically suppressed after co-incubation with L-NAME relative to the Clino group (Figure 3A, 3B, 3C). To quantify tube formation, the total tube length was calculated using Image-Pro Plus software. Co-incubation with L-NAME decreased HUVEC-C tube length by 252±17%, relative to the Clino group (Figure 3D). These findings suggest L-NAME significantly blocked simulated microgravity-enhanced HUVEC-C tube formation.

### Effects of L-NAME on the Stimulation of HUVEC-C Migration by Simulated Microgravity

**Figure 4:**
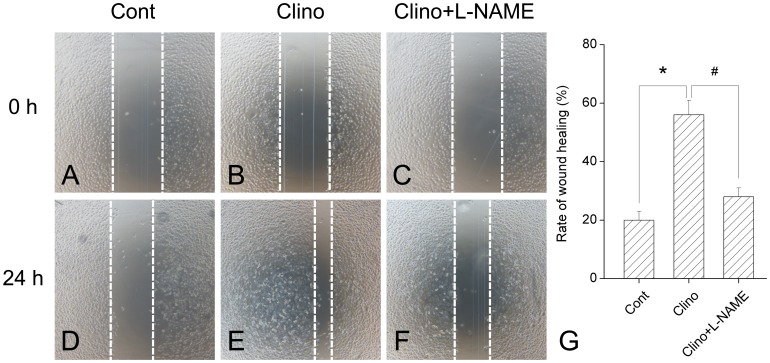
L-NAME inhibits simulated microgravity-induced HUVEC-C migration. An in vitro wound-healing assay was performed to evaluate the migration abilities of HUVEC-C treated with clinorotation in the absence or presence of L-NAME. The confluent HUVEC-C were wounded by scraping with a yellow tip. The cells were (A, B, C) washed, and then (D, E, F) incubated 24 h with fresh medium. The migration ability was determined by measuring the width of each denuded area and by calculating through the formula: 100%−(width _24 h_/width_ 0 h_) × 100%. (G): Result depicted that simulated microgravity caused an obviously increase in wound healing which was abrogated by L-NAME treatment. The data represent mean ± SD of three independent experiments. ^*^ *P*<0.05 versus control group; ^#^ *P*<0.05 versus clinorotation group.

Tube formation *in vitro* requires both cell attachment to extracellular matrix (ECM) and cell migration. Cell motility was assessed with a scratch assay, in which migration was initiated in a confluent layer of cells by mechanical denuding. A wound healing assay was performed to evaluate endothelial migration under simulated microgravity treatment. After culture under different conditions, HUVEC-C monolayers were mechanically wounded by scraping with a pipette tip (Figure 4A, 4B, 4C) and wound healing was observed 24 h later. Results showed that simulated microgravity caused a 180±9.6% increase in wound healing in comparison with control (Figure 4E *vs.* 4D). L-NAME inhibited the clinorotation-induced migration enhancement by approximately 79.2% (Figure 4F *vs.* 4E). These results indicated that L-NAME can also block simulated microgravity-induced endothelial cell migration.

### Effects of Simulated Microgravity on Regulation of eNOS

**Figure 5:**
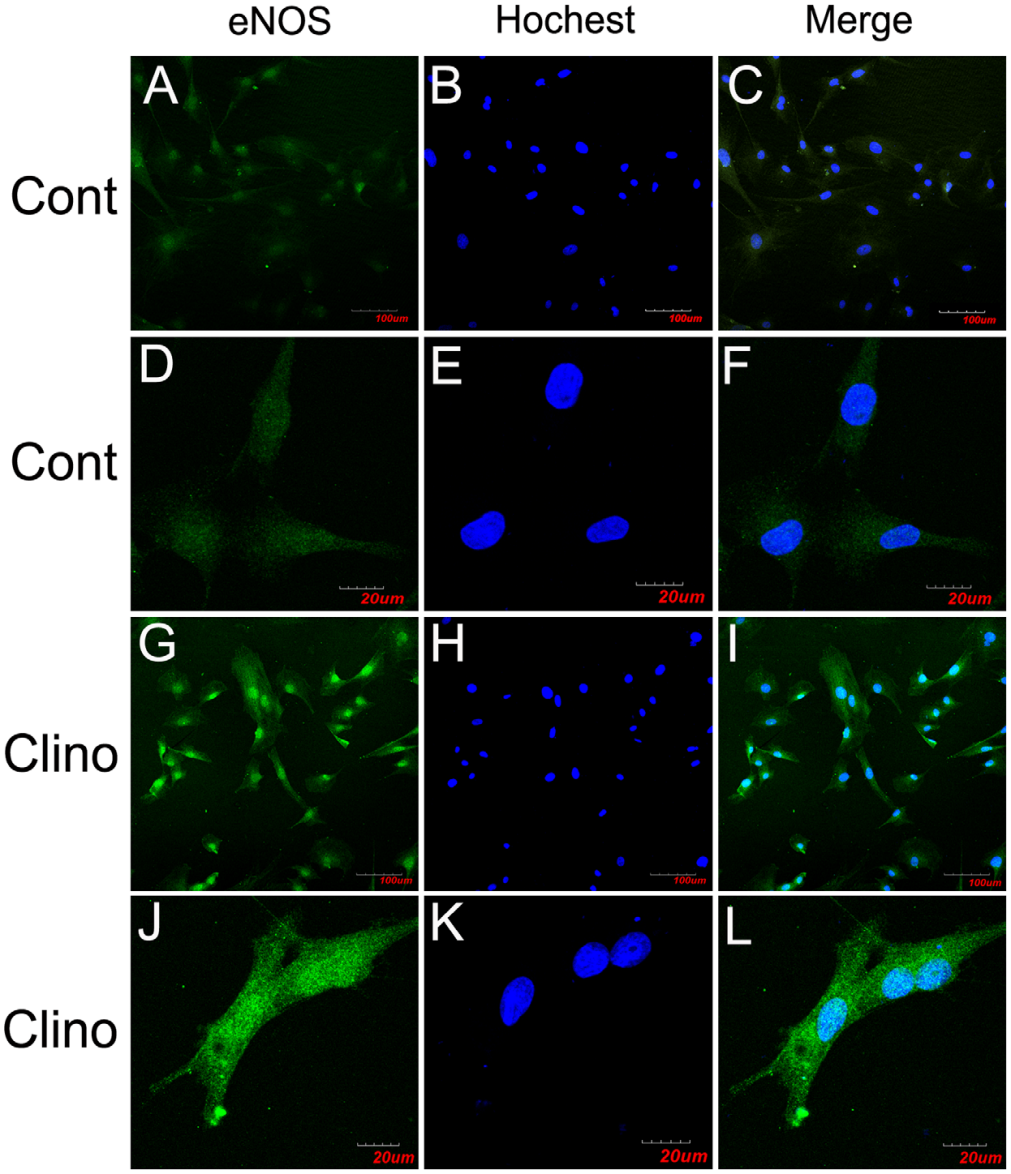
Simulated microgravity increases eNOS expression in HUVEC-C. Confocal microscopy localization of eNOS (Alexa-488, represented in green) in HUVEC-C exposed to 24 h of (A–F) normal gravity or to (G–L) simulated microgravity. Color images result from integrating different optical sections. The images in (A–C) and (G–I) were obtained by using the ×10 air objectives, and those in (D–F) and (J–L) were obtained by using the ×60 water immersion objectives. The images in (A–C) and (G–I) show high numbers of cells, but this in (D–F) and (J–L) show few cells.(C, F, I, L) Superposition of the green (eNOS) and blue (nucleus) signals.

In order to evaluate possible mechanisms of enhanced angiogenesis induced by simulated microgravity, we performed confocal microscopy analysis to study the effect of simulated microgravity on eNOS expression and localization in HUVEC-C. As shown in Figure 5, localization of eNOS in HUVEC-C by immunofluorescence revealed that the enzyme mostly accumulated in the cytoplasm near the nucleus and was also detected at the periphery of the cells. Clinorotation treatment slightly extended the eNOS immune-reactive area and simultaneously enhanced the intensity of eNOS immunofluorescence in HUVEC-C relative to controls. Analysis of 30 different sections indicates that 24 h of simulated microgravity increased eNOS expression in HUVEC-C.

### Effects of Simulated Microgravity on Caveolae Density in HUVEC-C

**Figure 6:**
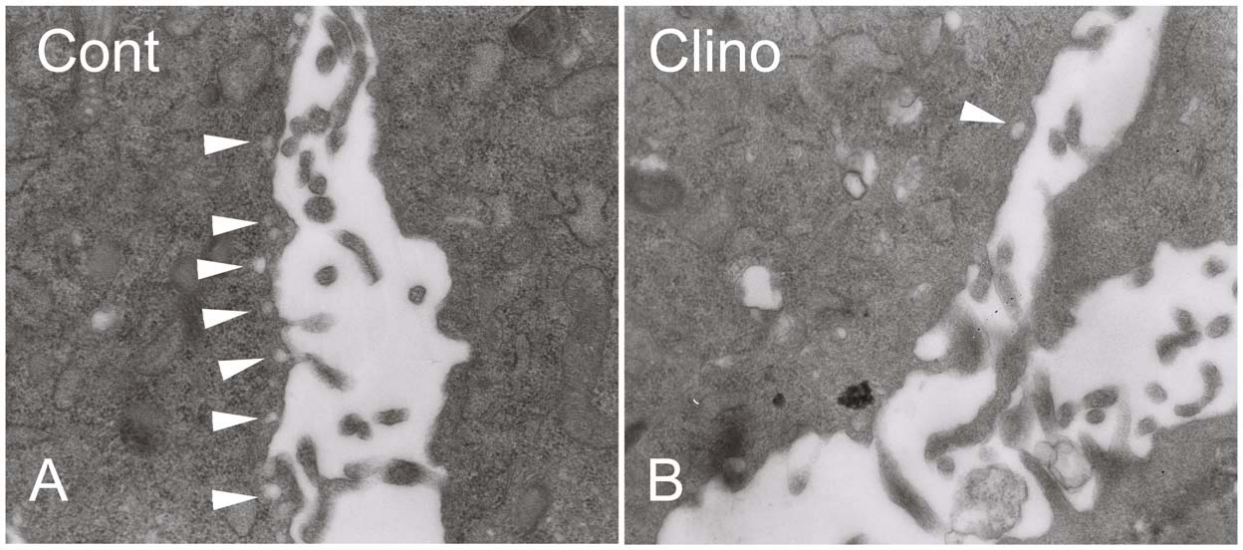
Simulated microgravity decreased the number of caveolae in HUVEC-C. Samples were fixed with glutaraldehyde, postfixed with osmium tetroxide, and stained with uranyl acetate and lead citrate. Electron micrographs showed views of the plasma membrane. Magnification ×30,000. A: Control cells; B: Clinorotation cells. Arrows denote representative caveolae organelles. Depending on the plane of the section, note that caveolae can appear as free vesicles or attached omega-shaped flasks.

Caveolae are unique lipid spheres in the plasma membrane that take part in the regulation of eNOS availability [28]. Transmission electron microscopy was used to observe the quantity and integrity of caveolae in the plasma membrane of each group HUVEC-C. Caveolae organelles were abundant in control HUVEC-C and could be distinguished by their characteristic size (50–100 nm), flask-like shapes, and location on or juxtaposition to the plasma membrane (Figure 6A). Every field of sectioned HUVEC-C examined in this way contained at least 5–10 caveolae. In contrast, clinorotation-treated HUVEC-C contained few if any detectable caveolae organelles. Despite viewing >10 fields of each of these cells, only one structure resembling a caveolae was observed in HUVEC-C exposed to simulated microgravity (Figure 6B).

### Effects of Simulated Microgravity on Upregulation of eNOS mRNA Expression in HUVEC-C

**Figure 7:**
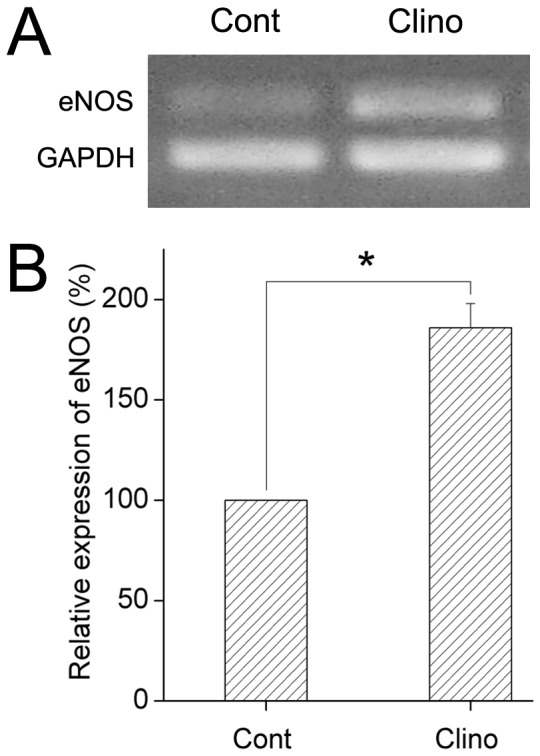
Simulated microgravity promoted the expression of eNOS mRNA in HUVEC-C. HUVEC-C were cultured under a static condition or exposed to clinorotation for 24 h. The eNOS mRNA levels were determined by RT-PCR in both groups, as described in Materials and Methods. Amplification of cDNA was performed in parallel samples using human GAPDH primers. Data are presented for expression of eNOS as a percentage change in band density normalized to GAPDH samples, and are shown as mean ± SD from 4 independent experiments. Results show that simulated microgravity significantly increase the eNOS mRNA level in HUVEC-C. ^*^ *P*<0.05 versus control.

To assess the influence of simulated microgravity on the expression of eNOS, we investigated eNOS mRNA using RT-PCR. HUVEC-C were cultured under static or clinorotation conditions for 24 h. As shown in Figure 7, the expression of eNOS mRNA remained low in the control group, while the level of eNOS mRNA was about 1.8-fold higher in the clinorotation group than in the control group (*P*<0.05). The data shown here indicated that 24 h clinorotation upregulate the eNOS mRNA expression in HUVEC-C.

### Mediation of the eNOS Activation Induced by Simulated Microgravity via the PI3K/Akt-dependent Signaling Pathway

**Figure 8:**
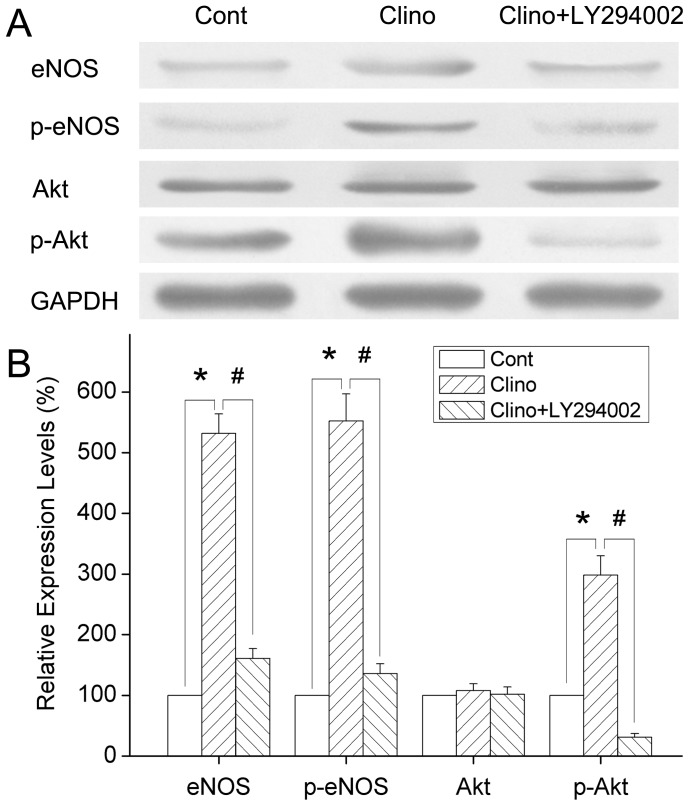
Effects of simulated microgravity on eNOS activity and role of the PI3K/Akt-dependent pathway in HUVEC-C. HUVEC-C were subjected to static culture and clinorotation in the presence or absence of LY294002, as described in Materials and Methods. The expression of total eNOS, Akt, and their phosphorylation were analyzed by Western blot. There was no significant change in total Akt protein expression between the control and clinorotation group. The expression of total eNOS was increased obviously after 24 h clinorotation. The phosphorylation of eNOS and Akt were upregulated after exposure to simulated microgravity, and clinorotation-induced changes were significantly suppressed by LY294002 treatment. Expressions were normalized to GAPDH. Results are expressed as percentages of the expression in control HUVEC-C. Values are mean±SD form 4 independent experiments. ^*^ *P*<0.05 versus control group; ^#^ *P*<0.05 versus clinorotation group.

To maintain the integrity of this study, the effect of simulated microgravity on eNOS protein expression was detected by Western blot analysis. The treatment with 24 h clinorotation increased the eNOS protein levels relative to controls (Figure 8). This shows that simulated microgravity can stimulate eNOS expression in HUVEC-C and that it may be a promoter of eNOS expression.

It has been shown that activation of the survival signal PI3K/Akt pathway and the endothelial specific eNOS/NO pathway is closely associated with vascular angiogenesis and remodeling [29], [30]. The PI3K/Akt signaling pathway can regulate eNOS activity, and activation of Akt has been reported to stimulate phosphorylation of eNOS. Therefore, to clarify the possible mechanisms underlying the activation of eNOS, we determined whether simulated microgravity can regulate phosphorylation of Akt and eNOS.

**Figure 9:**
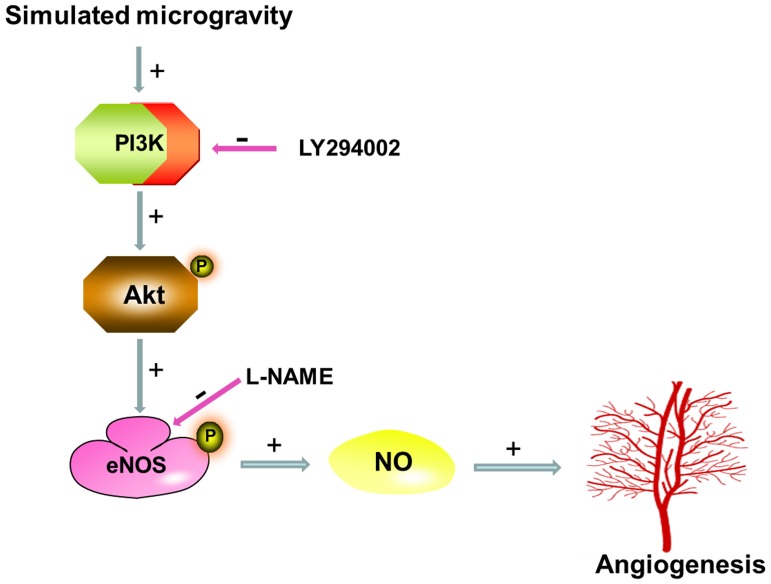
Role of PI3K-Akt-eNOS signaling cascade in simulated microgravity induced angiogenesis in HUVEC-C.

In order to evaluate this possibility, HUVEC-C were subjected to clinorotation in the presence or absence of LY294002 (a specific PI3K inhibitor). After exposure to simulated microgravity for 24 h, the expression of total eNOS and the amount of phosphorylated eNOS in HUVEC-C were both significantly increased (Figure 8). This is consistent with the idea that simulated microgravity activates eNOS. Western blot analyses also showed that the phosphorylated Akt were dramatically promoted (Figure 8). Intriguingly, treatment of HUVEC-C with LY294002 not only suppresses the phosphorylation of Akt but also downregulates the expression of eNOS and phosphorylated eNOS (Figure 8), suggesting that simulated microgravity induces the activation of eNOS via the PI3K/Akt signal pathway.

## Discussion

The most significant and novel of the findings discussed in this manuscript are that 1) simulated microgravity promoted angiogenesis and migration behaviors through the increased eNOS expression and that 2) the up-regulation of eNOS induced by simulated microgravity is mediated by means of PI3K-Akt pathway. In this way, our studies reveal a previously unrecognized role of simulated microgravity in endothelial cell angiogenesis. We suggest underlying mechanisms for its generation and function in this process and novel complementary explanations to microgravity-induced vascular remodeling.

Angiogenesis, a process by which new blood vessels are formed, plays an important role in physiological and pathological processes in humans. ECs located in the entire inner surfaces of the blood vessels are the main players in angiogenesis. It has been reported that ECs are highly sensitive to microgravity, including the alterations in morphology and gene expression. However, effects of microgravity on ECs angiogenesis are largely unknown. Recently, Infanger et al. demonstrated that human endothelial EA.hy926 cells that were exposed to simulated microgravity formed tube-like structures (TSs) within 48 h of detaching from the bottom of a culture flask [31]. Recently, they further described that these TSs consisted of several cell layers surrounding a central lumen. At the inner surface of the TSs, ECs were lined up and incorporated into abundant extracellular matrix so that the innermost layer of the TSs resembled the intima [32]. The findings led to the idea that culturing ECs under conditions of modeled gravitational unloading could become a new strategy for engineering small blood vessels. In addition, it could become a new method of studying neovascularization, because angiogenesis *in vivo* starts with the outgrowth of ECs, which assemble into tubes with tight cell-cell connections. Nevertheless, the angiogenic response of ECs to microgravity is still debatable. Morbidelli et al. showed that hypogravity conditions cause a marked impairment of ECs responsiveness to angiogenic factors and reduced their ability to migrate [6]. Due to heterogeneity in experimental approaches, such as variations in the duration of exposure, levels of the gravity and cell types tested, the inconsistency of these results was understandable.

There are great differences between in vivo condition and in vitro model with clinorotation to study the vascular endothelium changes induced by microgravity. Recent researches showed that an exposure to microgravity shifts the mean arterial pressure of the head from 70 mmHg in the upright posture on Earth to 100 mmHg in space, and the opposite alterations occurred in lower body vascular systems. The redistribution of blood flows induced by microgravity included changes of transmural pressures and shear stress within the arterial vasculature. Therefore, in microgravity environment, the alterations of endothelium in human were not only related to microgravity, but also to the hemodynamic changes in the cardiovascular and the imbalance of nervous-humoral regulation. To investigate the effects of simulated microgravity as a single stimulation on ECs function, we performed a series of experiments with clinorotation in order to eliminate hemodynamic influences. In this study, we carried out an in vitro tube formation and wound healing experiment to evaluate the effects of simulated microgravity on endothelial cell function. Our results showed that 24 h of clinorotation enhanced angiogenesis and migration in endothelium. Our data obtained here are in agreement with previous reports [33]. The cellular mechanisms involved in the alteration of endothelial functions induced by simulated microgravity are still not clearly understood.

NO, which is manufactured locally in the endothelium, is an important mediator of blood flow control, vascular permeability, and angiogenesis [34], [35]. eNOS-derived NO is a crucial contributor to the maintenance of cardiovascular homeostasis. The eNOS/NO pathway has been shown to exert a permissive role for angiogenesis in adult organisms [36], [37]. Inhibition of endogenously produced NO or disruption of the eNOS gene has been found to reduce new blood vessel formation in an animal model [30], [38], [39]. Both *in vivo* and *in vitro* studies have found that suppression of eNOS activation can inhibit angiogenesis [40]. In the present study, we evaluated the expression of the eNOS gene at the transcription and translation levels using RT-PCR, immunofluorescence staining, and Western blot analysis to determine whether eNOS activation is involved in the mechanisms underlying the potentiation of angiogenic responses. After 24 h of clinorotation, eNOS mRNA and protein expression were both significantly up-regulated. We then examined the angiogenesis after co-culturing with L-NAME, a specific inhibitor of eNOS. As expected, the tube formation and migration induced by simulated microgravity were dramatically suppressed in HUVEC-C.

Caveolae are specialized microdomains on the plasma membrane. They are involved in transcytosis and endocytosis, and a good body of evidence has shown that caveolae compartmentalize and integrate signaling events at the cell surface [41], [42]. A variety of protein signaling molecules involved eNOS are concentrated in caveolae. Caveolae membranes are characterized by a group of structural proteins called caveolins. Caveolin-1, a widely expressed isoform in ECs, has attracted much attention due to its ability to anchor eNOS. The binding of eNOS to caveolin-1 inhibits eNOS activity [28]. Many studies have documented caveolae as endogenous negative regulator of eNOS function [43]–[45]. Enzo and collaborators found that endothelial cell caveolae could constitute a mechanosensing system involved in hypergravity adaptation of human endothelial cells [46]. They also indicated that short microgravity exposure strongly affected eNOS activity associated with caveolin-1 (Tyr^14^) phosphorylation [47]. Therefore, they have proposed that one of the early molecular mechanisms responsible for gravity sensing of endothelium involves endothelial cell caveolae and caveolin-1 phosphorylation. In this study, we observed the ultrastructure of caveolae by transmission electron microscopy and found that 24 h of exposure to simulated microgravity not only impaired the structural integrity of caveolae but also dramatically decreased the number of them in the membrane of HUVEC-C. Decreases in caveolae density in the plasma membrane are correlated with enhanced activity of eNOS.

All of these results confirmed that up-regulation of eNOS was correlated with the stimulated angiogenesis of HUVEC-C under simulated microgravity conditions. The mechanisms underlying the up-regulation of eNOS were then investigated. It has been reported that in ECs, eNOS is phosphorylated by the Akt protein kinase, resulting in an increase in eNOS activity. It plays a crucial role in the regulation of vascular tone, vascular remodeling, angiogenesis, and NO production [48]. Early studies have suggested that the VEGF-induced increases in the release of NO release from ECsare attenuated by PI3K inhibitors and that VEGF stimulates Akt-mediated eNOS phosphorylation, leading to an increase in eNOS activity [49]. It also has been shown to protect ECs from serum-deprivation-induced apoptosis and to promote the formation of capillary-like structures on Matrigel in an Akt-dependent manner [48]. Dimmeler et al. reported that NO production in response to shear stress is controlled by the Akt-dependent phosphorylation of eNOS in cultured ECs [19]. Taken together, these findings suggest that PI3K activates Akt, which in turn is responsible for regulating the phosphorylation and activation of eNOS [50]. Therefore, to examine the functional involvement of Akt and eNOS in simulated microgravity-induced angiogenesis, we determined the effect of the chemical inhibitors of PI3K (LY294002) on simulated microgravity-induced signaling events. After exposure to clinorotation for 24 h, the phosphorylation of both Akt and eNOS was increased by approximately 2.9-fold and 5.4-fold, respectively, over control values. Co-culture with LY294002 not only downregulated the expression of phosphorylated Akt, but also downregulated eNOS phosphorylation, which suggested that the PI3K/Akt signal pathway participated in modulating the activity of eNOS. These experiments provide the first evidence that activation of PI3K-Akt-eNOS is a crucial event in the simulated microgravity-mediated signal transduction that leads to angiogenesis.

### Conclusion

The present study involves examination of the underlying mechanisms of angiogenesis in HUVEC-C after exposure to simulated microgravity via clinostat device. Our results suggest that 24 h of simulated microgravity can promote angiogenesis, migration, and eNOS expression in HUVEC-C. Further investigations have confirmed that the increased levels angiogenesis after simulated microgravity in HUVEC-C are mediated via PI3K-Akt-eNOS signal pathway (Figure 9). We plan a more detailed analysis of how microgravity affects the angiogenic process in ECs and we are currently investigating the precise molecular mechanisms responsible for the effects of simulated microgravity on the PI3K signaling pathway.
